# Transcription Factor NAC075 Delays Leaf Senescence by Deterring Reactive Oxygen Species Accumulation in *Arabidopsis*

**DOI:** 10.3389/fpls.2021.634040

**Published:** 2021-02-24

**Authors:** Chengcheng Kan, Yi Zhang, Hou-Ling Wang, Yingbai Shen, Xinli Xia, Hongwei Guo, Zhonghai Li

**Affiliations:** ^1^Beijing Advanced Innovation Center for Tree Breeding by Molecular Design, Beijing Forestry University, Beijing, China; ^2^National Engineering Laboratory for Tree Breeding, College of Biological Sciences and Technology, Beijing Forestry University, Beijing, China; ^3^Key Laboratory of Molecular Design for Plant Cell Factory of Guangdong Higher Education Institutes, Department of Biology, Southern University of Science and Technology (SUSTech), Shenzhen, China

**Keywords:** leaf senescence, NAC transcription factor, reactive oxygen species, catalase, *Arabidopsis thaliana*

## Abstract

Leaf senescence is a highly complex genetic process that is finely tuned by multiple layers of regulation. Among them, transcriptional regulation plays a critical role in controlling the initiation and progression of leaf senescence. Here, we found that the NAC transcription factor NAC075 functions as a novel negative regulator of leaf senescence. Loss of function of NAC075 promotes leaf senescence in an age-dependent manner, whereas constitutive overexpression of *NAC075* delays senescence in *Arabidopsis*. Transcriptome analysis revealed that transcript levels of antioxidant enzymes such as catalase (CAT), ascorbate peroxidase (APX), and superoxide dismutase (SOD) are significantly suppressed in *nac075* mutants compared with wild-type plants. Electrophoretic mobility shift assay (EMSA) and chromatin immunoprecipitation (ChIP) analyses revealed that NAC075 directly binds the promoter of *catalase 2* (*CAT2*). Moreover, genetic analysis showed that overexpression of *CAT2* suppresses the overproduction of reactive oxygen species (ROS) and the early senescence phenotypes of *nac075* mutants, suggesting that CAT2 acts downstream of NAC075 to delay leaf senescence by repressing ROS accumulation. Collectively, our findings provide a new regulatory module involving NAC075-CAT2-ROS in controlling leaf senescence in *Arabidopsis*.

## Introduction

Leaf senescence is a universal biological phenomenon in nature that contributes to the recycling of nutrients (Guo and Gan, 2005; Hughes and Reynolds, 2005). Senescence is the last stage of leaf development, accompanied by the hydrolysis of a series of macromolecules and the disassembly of chloroplasts and mitochondria, which ultimately leads to leaf death (Buchanan-Wollaston et al., 2005; Mao et al., 2017; Woo et al., 2019). Among them, yellowing of leaves from tip to base due to the loss of chlorophyll is the most striking marker of leaf senescence (Guo and Gan, 2005; Li et al., 2019; Woo et al., 2019). During leaf senescence, nutrients released by the catabolism of macromolecular substances such as proteins, lipids, and nucleic acids are transferred to active growing organs such as new buds and developing fruits and seeds, or stored for use in the next growing season (Guo and Gan, 2005; Lim et al., 2007; Woo et al., 2019). Efficient senescence is essential for maximizing viability in the next generation or season, while premature senescence induced by a variety of environmental factors declines crop plants’ yield and quality (Breeze et al., 2011). Thus, the appropriate onset and progression of leaf senescence are essential for plant fitness, suggesting that senescence evolves as a life history strategy. Significant advances in understanding the regulatory mechanisms of leaf senescence will provide valuable clues for the manipulation of traits of agronomical important plants.

Leaf senescence is a highly complex and orderly dynamic regulation process and is strictly controlled by multiple layers of regulation, including chromatin-mediated, transcriptional, post-transcriptional, translational, and post-translational regulation (Woo et al., 2013, 2019; Kim et al., 2016, 2018). Leaf senescence is not a passive but a highly coordinated process that is regulated by a number of senescence-associated genes (*SAG*s), whose transcripts increase as leaves age. The onset, development, and completion of leaf senescence involve extensive regulation of gene expression (Woo et al., 2013). Genome-wide transcriptional analysis revealed that the leaf senescence process is accompanied by differential expression of thousands of *SAG*s (Breeze et al., 2011; Woo et al., 2016). At the transcription level, transcription factors (TFs) act as core control elements to drive the drastic changes in *SAG*s expression during leaf senescence. The dynamic activation of TFs is triggered by internal signals such as plant hormones or environmental factors such as high salt (Guo and Gan, 2005; Guo, 2013; Li et al., 2018). Significant advances in dissecting the regulatory mechanisms underpinning leaf senescence have benefited from the identification and functional assessment of hundreds of SAGs and their corresponding mutants. Previous studies have identified numerous TFs that participate in the process of leaf senescence in *Arabidopsis*, including NAC (NAM, ATAF1, 2, and CUC2), WRKY, MYB, and bZIP families’ TFs, which play important roles in regulating leaf senescence (Lim et al., 2007; Balazadeh et al., 2008; Woo et al., 2019). As one of the largest TF families in plants, NAC TFs receive widespread attention due to their important role in regulating leaf senescence process in a variety of plant species (Kim et al., 2016). The regulatory roles of a number of NAC TFs in leaf senescence have been characterized in *Arabidopsis*. For instance, ORE1 (ANAC092), AtNAP (ANAC029), ATAF1 (ANAC002), JUB1 (ANAC042/ANAC2), VNI2 (ANAC083), and ANAC017/082/090 act as positive or negative regulators of leaf senescence (Guo and Gan, 2006; Balazadeh et al., 2010; Yang et al., 2011; Wu et al., 2012; Jensen et al., 2013; Garapati et al., 2015; Kim et al., 2018). Recent findings revealed that the molecular network of NAC TFs regulates leaf senescence by integrating internal developmental signals and numerous environmental signals (Kim et al., 2016). Although a growing body of evidence indicates that NAC TFs play important roles in leaf senescence, little is known regarding their importance and underlying regulatory mechanisms.

In this study, we found that NAC TF NAC075 functions as a negative regulator of leaf senescence. Mutation of NAC075 evidently hastens leaf senescence, whereas overexpression of *NAC075* markedly prolongs leaf longevity. Biochemical and genetic evidence shows that NAC075 delays leaf senescence by directly upregulating *CAT2* expression and suppressing the accumulation of reactive oxygen species (ROS) in *Arabidopsis*.

## Materials and Methods

### Plant Materials and Growth Conditions

The *Arabidopsis thaliana* ecotype Columbia (Col-0) is the parent strain for all mutants and transgenic lines used in this study. The transfer DNA (T-DNA) insertional mutant *nac075* (SALK_132120C) was obtained from the Nottingham Arabidopsis Stock Centre (NASC). The *nac075 CAT2ox* was generated by genetic cross, and the homozygous plants were identified through PCR-based genotyping. Seeds were surface-sterilized and plated on Murashige and Skoog (MS) medium (4.3 g/L MS salts, 1% sucrose, pH 5.7–5.8, and 8 g/L agar). After stratifying at 4°C for 3 days to improve germination uniformity, the plates were transferred to an environmentally controlled growth room (PAR of 100–150 μE m^–2^ s^–1^) for 4 days. For plant leaf senescence phenotypic analysis, light-grown seedlings were transferred to soil and grown at 22°C under long-day conditions (16-h light/8-h dark).

### Plasmid Construction and Generation of Transgenic Plants

To construct *ProNAC075:GUS*/Col-0, a 3-kb genomic promoter sequence was amplified and inserted into pCambia1391 vector (GenBank Accession-AF234308). To generate *35S:GFP-NAC075*/Col-0, the full-length *NAC075* CDS sequence was amplified and then inserted into pEGAD vector (Cutler et al., 2000). To generate inducible overexpressing lines, the full-length *NAC075* CDS fused with 3xFLAG was into pER8 vector (Zuo et al., 2000). All constructs were transformed into *Agrobacterium tumefaciens* cells (strain GV3101), which was used to transform Col-0 plants by the floral dip method (Clough and Bent, 1998). Primers used for PCR are listed in Supplementary Table 1.

### RNA Isolation and Real-Time PCR Analysis

Total RNA was isolated by using plant RNA extraction kits (ER301; TransGen Biotech, China), and the complementary DNA was produced using TransScript All-in-One First-Strand cDNA Synthesis kit (AT341; TransGen Biotech). Transcript levels were detected with TransStart Green qPCR SuperMix (AQ111; TransGen Biotech) by using Applied Biosystems 7500 Real-Time PCR System (Life Technologies, Carlsbad, California, United States). Ubiquitin-conjugating enzyme 21 (*UBC21*, AT5G25760) was used as an internal control to normalize the gene expression level. The primers used in this study are listed in Supplementary Table 1.

### Measurement of Chlorophyll Contents and Maximal Photochemical Efficiencies of PSII

Chlorophyll contents were measured in the third and fourth rosette leaves of *Arabidopsis* using a chlorophyll meter Konica Minolta SPAD502 Plus (Sakura-machi, Hino-shi Tokyo, Japan), and three biological replicates were performed. Maximal photochemical efficiencies of Photosystem II (PSII, Fv/Fm) were measured by using a MultiSpeQ instrument (East Lansing, MI, United States).

### GUS Staining

GUS (β-Glucuronidase) staining was performed as described previously (Jefferson, 1989). Plant tissues were incubated with GUS staining solution (100 mM Na_3_PO_4_, pH 7.0, 1 mM EDTA, 1 mM potassium ferrocyanide, 1 mM potassium ferricyanide, 1% Triton X-100, and 1 mg/ml 5-bromo-4-chloro-3-indolyl-β-D-glucuronide) for 8 to 12 h at 37°C in the dark, followed by decolonization using 95% ethanol.

### Detection of Hydrogen Peroxide and Superoxide

The third and fourth leaves (18-day-old) were vacuum-infiltrated with diaminobenzidine tetrahydrochloride (DAB) solution (1 mg/ml 3,3’-diaminobenzidine-4HCl, pH 3.8) and NBT (nitroblue-tetrazole) solution (0.5 mg/ml NBT, 10 mM potassium phosphate, pH 7.8, and 10 mM sodium azide) to detect hydrogen peroxide and superoxide, respectively, incubated in the dark for 8–10 h, and decolorized in 95% ethanol. The intensity of brown and blue coloration indicates H_2_O_2_ and O^2–^ contents, respectively.

### Trypan Blue Staining

Trypan blue staining was performed as described previously with minor modifications (Kim et al., 2009). Briefly, the third and fourth rosette leaves were soaked in trypan blue staining solution (10 g phenol, 10 ml glycerol, 10 ml lactic acid, 10 ml ddH_2_O_2_, and 0.02 g trypan blue) and stained in a boiling water bath for 3–5 min. Three leaves were completely submerged in trypan blue staining solution. After leaving overnight at room temperature, the leaves were carefully clamped into the decolorizing solution (2.5 g/ml chloral hydrate).

### RNA-Sequencing Analysis

The third and fourth rosette leaves of 24-day-old Col-0 and *nac075* mutant plants were collected and ground into a powder in liquid nitrogen. Total RNA was extracted using an RNeasy Plant kit (Qiagen), and the quality and quantity of RNA were detected using an IMPLEN NanoPhotometer (GmbH). RNA-seq data were generated with an Illumina HiSeq 2000 system at Biomarker Ltd. (Beijing, China). Raw reads (fastq format) were trimmed and filtered through in-house perl scripts (Biomarker Ltd. China). The reads were then mapped to *Arabidopsis* reference genome using Hisat2 algorithm. DEGs were filtered using the following criteria: | Log2 (fold change)| > 1.0, *P* < 0.05. Gene ontology (GO) enrichment analysis was performed by using the GO database^1^. Default parameters were used for all bioinformatics software. Raw RNA-seq reads are available at the National Center for Biotechnology Information^2^.

### Electrophoretic Mobility Shift Assay (EMSA)

The full-length coding region of NAC075 was produced by quantitative RT-PCR (qRT-PCR) and used for developing the DNA constructs to pET32a for the expression of recombinant proteins in *Escherichia coli* BL21 (DE3). Purification of NAC075 protein was conducted according to the protocol included with the His-Trap HP pre-packed minicolumns (GE Healthcare Life Sciences, Uppsala, Sweden). EMSA was performed according to the user guide from the LightShift Chemiluminescent EMSA Kit (Lot#20148, Thermo Scientific). Briefly, the binding reaction was performed in a total volume of 20 μl by incubation of an appropriate amount of NAC075 proteins with 20 fm of biotin-labeled probe DNA and 1 μg of poly (dI–dC) in a reaction buffer (25 mM HEPES-KOH, pH 7.5, 100 mM KCl, 0.1 mM EDTA, 10% [v/v] glycerol, and 1 mM DTT) at room temperature for 30 min. The binding reaction products were resolved on a 6% polyacrylamide gel run in 0.5 × TBE buffer. 5’-biotin-labeled oligonucleotide of *CAT2* was synthesized and used as probes in EMSA (Supplementary Table 1).

### Chromatin Immunoprecipitation (ChIP) Assays

ChIP experiments were performed as described previously with minor modifications (Saleh et al., 2008). Briefly, 5 g of 4-week-old *35S:GFP-NAC075* leaves was collected into 50-ml Falcon tubes with 37 ml of cross-linked buffer (10 mM Tris–HCl, pH 8.0, 0.4 M sucrose, 1 mM EDTA, 1 mM PMSF, and 1% formaldehyde). Next, 2 M glycine was added for 5 min to quench the cross-linking reaction. The leaves were then washed three times with sterile deionized water, frozen in liquid nitrogen, and quickly ground into a powder. Next, the ground powder was added into 25 ml of nuclear separation buffer and vortexed to isolate chromatin DNA. The sonicated chromatin supernatant (300 μl) was diluted and 50 μl of salmon sperm DNA/protein A agarose beads (Upstate) was added for pre-clearing at 4°C for 1 h with gentle rotation (12 rpm). The solutions were then transferred into two new tubes. Add 10 μl of anti-GFP monoclonal antibody with a dilution of 1:150 (v/v) to one tube, but not the other (as a negative control). After incubation at 4°C overnight, beads were washed with low-salt wash buffer, high-salt wash buffer, and Tris–EDTA (TE) buffer, followed by followed by Proteinase K (10 mg/ml; Sigma-Aldrich) treatment and reverse cross-linking with 5 M NaCl. DNA was extracted with phenol/chloroform/isoamyl alcohol (25:24:1), and then ethanol precipitated with 2 μl of 20 mg/ml glycogen. The purified DNA was resuspended in 20 μl of distilled water and stored at -20°C. All oligonucleotide sequences used here are listed in Supplementary Table 1.

### Plant Hormone-Induced Leaf Senescence

The third and fourth leaves (20-day-old) detached from Col-0, *nac075*, and *NAC075ox* plants were treated with 5 mM MES (Mock), 10 μM ACC, 50 mM MeJA, 1 mM SA, 50 μM ABA, 10 mM H_2_O_2_, or 100 mM NaCl in dark conditions for 3 days, respectively.

### Induction of *NAC075* Gene Expression by Treatment With β-Estradiol

The 28-day-old *pER8-FLAG-NAC075* transgenic plants were sprayed with 50 μM β-estradiol. After treatment for 0.5, 1, and 4 h, the third and fourth rosette leaves were detached and used for RNA extraction and qRT-PCR analysis.

## Results

### Transcript Level of *NAC075* Increases as Leaf Ages

**FIGURE 1:**
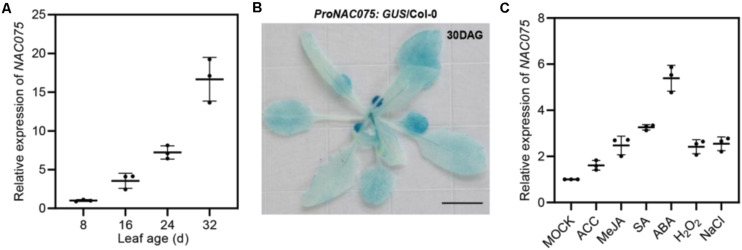
NAC075 transcription factor is a senescence-associated gene that is induced by age, plant hormones, ROS, and salinity. **(A)** qRT-PCR analyses of *NAC075* expression in the third or fourth rosette leaves at the indicated leaf age. Data are represented as means ± SD (*n* = 3). **(B)** GUS staining of rosette leaves of 30-day-old plants harboring the GUS transgene driven by the promoter of *NAC075* (*ProNAC075:GUS*/Col-0). Scale bar, 1 cm. **(C)** qRT-PCR analyses of *NAC075* expression in the leaves of 20-day-old Col-0 plants upon treatment with 5 mM MES (Mock), 10 μM ACC, 50 mM MeJA, 1 mM SA, 50 μM ABA, 10 mM H_2_O_2_, or 100 mM NaCl for 6 h. Data are represented as means ± SD (*n* = 3).

Previous studies have shown that NAC TF NAC075 is related to leaf senescence (Woo et al., 2016; Li et al., 2020), but the underlying regulatory mechanism remains unclear. Toward this end, we firstly performed Real-Time Quantitative Reverse Transcription PCR (qRT-PCR) to examine the transcript levels of *NAC075* in *Arabidopsis* leaves at young, mature, early, and late stage of senescence (Figure 1A). Time-course analysis of mRNA level monitored by qRT-PCR revealed that the transcript level of *NAC075* gradually increased during leaf development and senescence (Figure 1A). *SAG12*, a widely used marker gene of leaf senescence (Noh and Amasino, 1999; Pontier et al., 1999), was specifically expressed in the senescing leaves (Supplementary Figure 1A). We also measured the transcript levels of *AtNAP* and *ORE1*, two well-known positive regulators of leaf senescence (Guo and Gan, 2006; Kim et al., 2009), and found that their expressions also increased as leaves age (Supplementary Figures 1B,C). To further verify the age-dependent regulation of *NAC075* mRNA *in planta*, we generated transgenic *Arabidopsis* expressing GUS (β-glucuronidase gene) driven by *NAC075* promoter containing a 3-kb fragment upstream promoter of the start codon (*ProNAC075:GUS/*Col-0). Histochemical staining assay of rosette leaves of 30-day-old *ProNAC075:GUS*/Col-0 plants revealed that the old yellowing leaves displayed higher GUS activity than that in young green leaves (Figure 1B), indicative of an increase in *NAC075* expression level during the leaf senescence process.

We next performed qRT-PCR to investigate the influences of other senescence-regulating signals such as plant hormones, ROS, and salt stress on the transcription of *NAC075*. We found that treatment with the ethylene precursor ACC (1-aminocyclopropane-1-carboxylate), methyl jasmonate (MeJA), salicylic acid (SA), abscisic acid (ABA), H_2_O_2_, and salt evidently increased the expression levels of NAC075 compared to the mock-treated plants (Figure 1C). Among them, treatment with ABA greatly enhanced *NAC075* transcription (Figure 1C), suggesting that NAC075 may be involved in ABA-induced leaf senescence process.

### NAC075 Negatively Regulates Leaf Senescence

**FIGURE 2:**
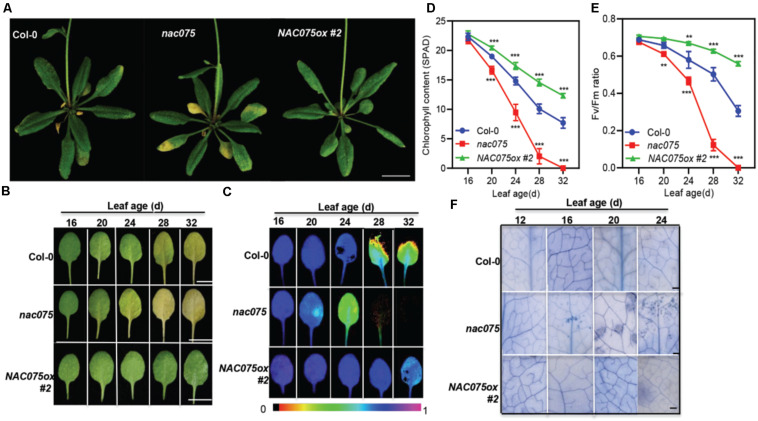
Age-dependent senescence symptoms in the *nac075* mutants and *NAC075ox* plants. **(A)** The senescence phenotypes of 30-day-old Col-0, *nac075* mutants, and *NAC075ox* plants. Early onset of leaf senescence in *nac075* mutant was observed compared with Col-0 plants grown under long-day condition. Scale bar, 1 cm. **(B)** The age-dependent leaf senescence phenotype of Col-0, *nac075* mutants, and *NAC075ox* plants. Photographs show the third or fourth rosette leaves at the indicated days after emergence (DAE). Scale bar, 1 cm. **(C)** Analysis of Fv/Fm in Col-0, *nac075* mutants, and *NAC075ox* plants as leaves age. Image generation was performed by IMAGING-PAM. Image processing was performed by Imaging Win software. **(D,E)** Chlorophyll content **(D)** and Fv/Fm **(E)** in Col-0, *nac075* mutants and *NAC075ox* plants as leaves age. Error bars indicate SD (*n* = 3). Student’s *t* test, ^∗∗^*P* < 0.01, ^∗∗∗^*P* < 0.001. **(F)** Trypan blue staining of leaves at the indicated leaf age. In each plant leaf, dead or dying leaf areas formed blue-colored patches of cells by trypan blue staining. Bar = 500 μm.

To investigate the function of NAC075 in leaf senescence, we examined the senescence-associated phenotypes of *nac075* knockout mutant carrying a T-DNA insert in the third intron of *NAC075* (Supplementary Figure 2A) and the transgenic plants overexpressing *NAC075* (*NAC075ox*) (Figure 2A). Genotyping analysis demonstrated that *nac075* mutant is a null allele (Supplementary Figure 2B), which was confirmed further by gene expression analysis (Supplementary Figure 2C). We performed qRT-PCR to detect the *NAC075* transcription in three *NAC075ox* lines (#1, #2, and #3), and selected *NAC075ox* #2 with the highest expression level for subsequent experiments (Supplementary Figure 3). We found that *nac075* mutant exhibited an early-senescence phenotype in comparison to Col-0 plants under long-day conditions (Figure 2A), while *NAC075ox* plant displayed a delayed senescence phenotype (Figure 2A), suggesting that NAC075 is a negative regulator of leaf senescence. Interestingly, the *nac075* mutants also displayed early silique senescence (Supplementary Figure 4), suggesting that NAC075 is also involved in silique development. Next, we examined the senescence characteristics of single leaf at different ages. Leaf yellowing occurred in the third or fourth rosette leaves of *nac075* mutant plants at 24 days after emergence (DAE), whereas the leaves of Col-0 and *NAC075ox* plants remained green. At 28 DAE, the third and fourth rosette leaves of *nac075* mutants were completely yellowed, which was not observed in Col-0 and *NAC075ox* plants (Figure 2B). At 32 DAE, the leaves of Col-0 plant began to turn yellow, while the leaves of *NAC075ox* plants remained green. Leaf yellowing caused by chloroplast decomposition and chlorophyll loss are typical characteristics of leaf senescence (Woo et al., 2001). We also monitored the chlorophyll contents, and photochemical efficiency of photosystem II (PSII; Fv/Fm) decreased more quickly and evidently in *nac075* mutant than in Col-0 (Figures 2C–E). The *NAC075ox* plants displayed delayed leaf senescence phenotypes, with elevated chlorophyll content and Fv/Fm compared with Col-0, demonstrating further that NAC075 is a negative regulator of leaf senescence. We also found that aging-induced cell death was accelerated in the *nac075* mutants, which was delayed in *NAC075ox* plants, as shown by the earlier emergence of trypan blue-stained cells in 16 and 24-day-old leaves (Figure 2F). Thus, NAC075 is a negative regulator of aging-induced cell death and senescence in *Arabidopsis* leaves.

Given that *NAC075* transcription was induced by multiple plant hormones and stresses (Figure 1C), we examined whether NAC075 is involved in the leaf senescence process triggered by these factors. To this end, the third or fourth rosette leaves of 20-day-old Col-0, *nac075* mutants, and *NAC075ox* plants were treated with darkness, plant hormones, ROS, and salt. We found that the leaves of *NAC075ox* plants exhibited the delayed senescence phenotypes upon treatment with these factors (Supplementary Figure 5), suggesting that NAC075 delays the leaf senescence process caused by numerous factors. In contrast, the senescence phenotype of *nac075* leaves was not evidently different from that of the wild-type Col-0, indicative of the existence of the functional redundancy among NAC TFs (Supplementary Figure 5).

### NAC075 Regulates Genes Involved in ROS Scavenging Processes

**FIGURE 3:**
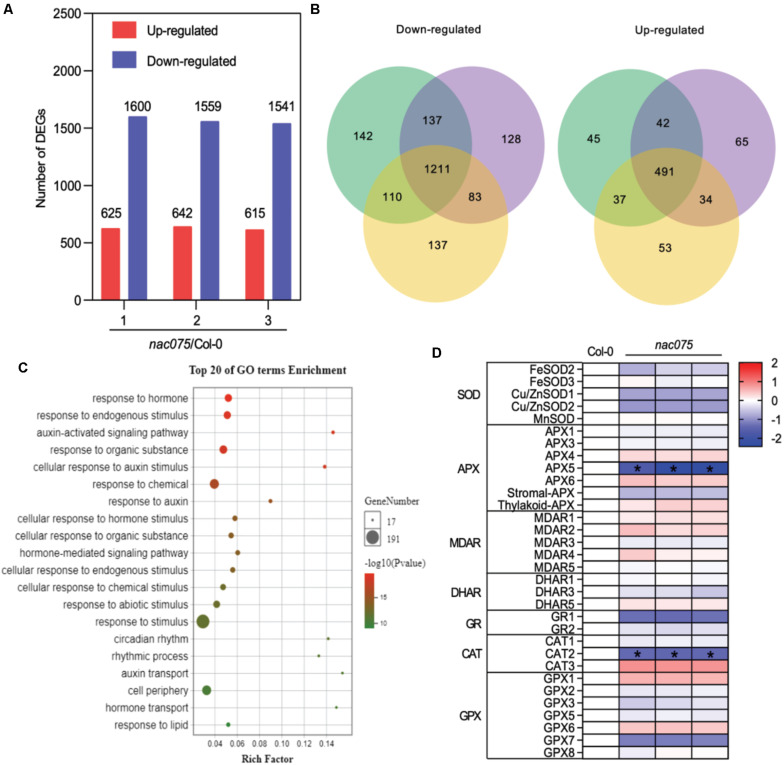
Genome-wide transcriptome analysis of *nac075* mutant plants. **(A)** The number of up-regulated and down-regulated genes in three biological replicates of *nac075* mutant plant rosette leaves vs. Col-0 with RNA-seq. Red represents up-regulated genes, and blue represents down-regulated genes. **(B)** Schematic of the Venn diagram analysis of common down-regulated and up-regulated genes [DEG; | Log2 (FC)| > 1, FDR < 0.05] in three *nac075* mutant plant leaves relative to the Col-0 control. **(C)** GO bubble diagram of down-regulated genes among the DEGs in three *nac075* rosette leaf samples. The bubble size represents the number of DEGs, and the bubble color represents the *P-*value. The rich factor equals the number of DEGs in a certain signaling pathway. **(D)** Heat map showing ROS-related genes in *nac075* mutant plant rosette leaves compared to Col-0. Means of three experiments are shown. The log2 fold change scale is indicated on the right side of the heat map. SOD, superoxide dismutase; APX, ascorbate peroxidase; MDAR, monodehydroascorbate reductase; DHAR, dehydroascorbate reductase; GR, glutathione reductase; CAT, catalase; GPX, glutathione peroxidate.

To elucidate the underlying mechanisms of NAC075 in the regulation of leaf senescence, we performed genome-wide mRNA expression analysis of wild-type and mutant leaves at the presenescent stage (24-day-old) to identify the candidate target genes. We compared the transcriptomes of wild-type leaves with those of *nac075* mutants and identified 2225, 2241, and 2156 differentially expressed genes (DEGs) in three biological replicates, respectively (Figure 3A). Out of them, 1721 genes (491 up-regulated genes and 1211 down-regulated genes) exhibited overlapping differential expression in three biological replicate samples (Figure 3B), suggesting substantial regulation by NAC075.

Next, in order to determine the cellular processes associated with the DEGs, we carried out enrichment analysis of GO biological processes (GOBPs) by subjecting the sequences to GO annotations (Young et al., 2010). Interestingly, the GOBP-association analysis revealed that responses to stimulus or chemical (such as salt stresses) and responses to oxygen-containing compounds (such as oxidative/ROS) are the top senescence-promoting processes regulated by NAC075 among all processes (Figure 3C). Overproduction of ROS caused by various stresses has been demonstrated as potentially critical for induction and maintenance of senescence in animals and plants (Woo et al., 2013). Therefore, we performed GO analysis on seven types of ROS-clearance genes in *DEG*s. The heat map showed that several ROS-clearance genes, such as *APX5* and *CAT2*, were down-regulated in mutants (Figure 3D), which is consistent with the early-senescence phenotype of *nac075* mutants (Figure 2). Collectively, these data suggest that NAC075 delays leaf senescence process through negatively regulating senescence-promoting processes such as responses to oxidative/ROS stress.

### NAC075 Directly Binds the *CAT2* Promoter to Activate Its Transcription

**FIGURE 4:**
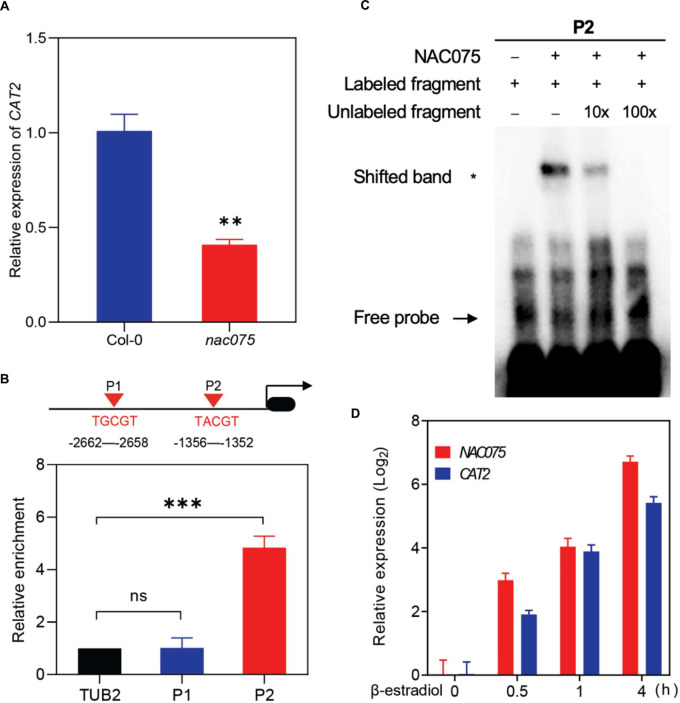
*CAT2* is a direct target of NAC075. **(A)** Relative expression level of *CAT2* in the third and fourth rosette leaves of 25-day-old Col-0 and *nac075* mutant plants. Data are means ± SD, *n* = 3. **(B)** NAC075 directly binds to the promoter of *CAT2*. Schematic diagram indicates the locations of two putative NAC075 binding sites (P1 and P2) in the *CAT2* promoter (top). ChIP-qPCR was performed to examine the relative NAC075 binding to the *CAT2* promoter (bottom). An anti-GFP monoclonal antibody was used for DNA immunoprecipitation from 28-day-old *Pro35S:NAC075-GFP*/Col-0 transgenic plants. The relative enrichment of NAC075 binding to *CAT2* promoter was normalized to *TUBULIN2* (*TUB2*). Student’s *t-*test, ^∗∗^*P* < 0 .01, ^∗∗∗^*P* < 0.001, ns, not significant. Data are means ± SD of three independent biological replicates. **(C)** EMSA assay of the binding of NAC075 to the *CAT2* promoter *in vitro*. Biotin-labeled probe was used to EMSA experiment and non-labeled fragments were used as competitors. The + and − symbols represent the presence and absence of components. Three biological replicates were performed with similar results. **(D)** qRT-PCR analysis of *NAC075* and *CAT2* expressions in the *pER8-FLAG-NAC075* plants treated with 50 mM β-estradiol for the indicated time points. Data are represented as means ± SD, *n* = 3.

The above data pushed us to explore whether NAC075 directly regulates expressions of genes related to ROS clearance. Interestingly, transcripts of *CATALASE2* (*CAT2*), an important ROS scavenging enzyme, were significantly decreased in the leaves of *nac075* mutants in comparison with Col-0 (Figure 4A), which is consistent with the transcriptome data (Supplementary Dataset 1). To examine whether *CAT2* is a direct target of NAC075, we firstly identified the NAC075 binding sites (NBSs) in the promoter regions of *CAT2*. Based on a previous study (Lindemose et al., 2014), two NBSs (TG/ACGT) were identified and then used for ChIP assay using 35-day-old *Pro35S:NAC075-GFP*/Col-0 (*NAC075ox*) transgenic plants. ChIP-qPCR analysis showed that NAC075 is significantly enriched in TACGT regions, indicating that NAC075 binds to these regions *in vivo* (Figure 4B). We next performed EMSAs to examine the *in vitro* binding activity of NAC075. The results revealed that NAC075 protein tagged with His (NAC075-His) was capable of binding probes containing P2, while it was unable to bind probes containing P1 (Figure 4C). Using unlabeled probes as competitors, competitive binding assays were carried out to confirm the binding specificity by adding an excess of unlabeled competitor DNA fragments (Figure 4C), suggesting further that NAC075 directly binds the promoter of *CAT2*. To further investigate the regulatory roles of NAC075 on *CAT2*, we generated inducible overexpressing plants *pER8-FLAG-NAC075*. Upon treatment with β-estradiol, *NAC075* transcripts increased (Figure 4D). As expected, expression levels of *CAT2* also increased (Figure 4D). Collectively, the above data reveal that NAC075 can directly bind the promoter of *CAT2* and regulate its expression.

### Overexpression of *CAT2* Suppresses the Early Senescence Phenotype of *nac075* Mutants

**FIGURE 5:**
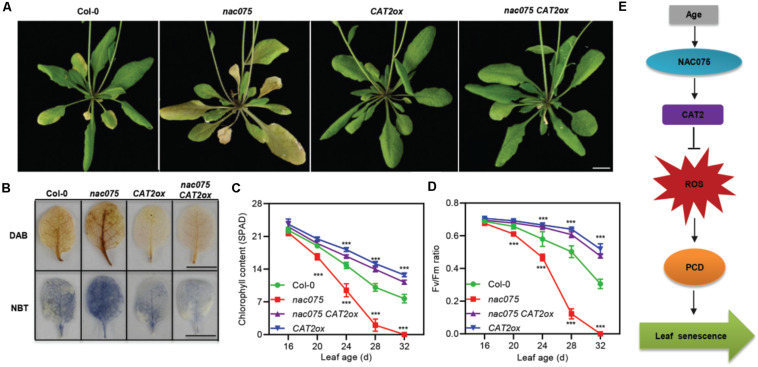
Overexpression of *CAT2* suppresses the early senescence phenotype of *nac075* mutant plants. **(A)** Leaf senescence phenotype of 32-day-old Col-0, *nac075*, *nac075 CAT2ox*, and *CAT2ox* plants. The scale bar represents 1 cm. **(B)** DAB and NBT staining were used to detect H_2_O_2_ and O^−^_2_ accumulation, respectively, in the third or fourth leaves of Col-0, *nac075*, *nac075 CAT2ox*, and *CAT2ox* plants. The brown and blue color represent H_2_O_2_ and O^−^_2_ accumulation, respectively. Scale bar, 1 cm. **(C,D)** Measurements of chlorophyll contents **(C)** and photochemical efficiency (Fv/Fm) **(D)** in Col-0, *nac075*, *nac075 CAT2ox*, and *CAT2ox* plants as leaves age. Data are represented as means ± SD, *n* = 3. The experiment was performed three times with similar results. Student’s *t-*test, ***P < 0.001. **(E)** A proposed model illustrates the transcription factor NAC075 that delays leaf senescence by deterring reactive oxygen species accumulation in *Arabidopsis*. NAC075 promotes *CAT2* transcription by directly binding to its promoter, which is able to suppress ROS overproduction. Decreased ROS levels are capable of reducing programmed cell death, thereby delaying leaf senescence.

To further explore the genetic regulatory relationship between NAC075 and CAT2 in leaf senescence, we generated *nac075 CAT2ox* plants by crossing the *CAT2ox* (Guo et al., 2017) transgenic plants to *nac075* mutant. Under long-day conditions, plants with the combined *nac075 CAT2ox* genotype showed an obvious delayed senescence phenotype compared with *nac075* mutant plants, indicating that the accelerated leaf senescence phenotype of *nac075* mutant plants was effectively suppressed by CAT2 (Figure 5A). Moreover, DAB staining showed that H_2_O_2_ levels in the *nac075 CAT2ox* and *CAT2ox* plants was significantly lower than that of *nac075* and Col-0 plants (Figure 5B). This indicates that overexpression of *CAT2* suppresses the elevated H_2_O_2_ levels in *nac075* mutant plants, which is in line with the observation that *CAT2* is the downstream target gene of NAC075. Similarly, higher chlorophyll content and Fv/Fm further confirmed the delayed senescence phenotype of *nac075 CAT2ox* plants (Figures 5C,D). Taken together, these results reveal that overexpression of *CAT2* suppresses the early senescence phenotype in *nac075* mutant plants by reducing H_2_O_2_ accumulation. Therefore, a regulatory module is proposed, which is the NAC075-CAT2 pathway modulates leaf senescence by regulating ROS levels (Figure 5E).

## Discussion

Leaf senescence is a process of programmed cell death (PCD), which not only is affected by various internal and external factors but also involves highly complex regulatory processes with the coordinated actions of multiple pathways (Lim et al., 2007; Woo et al., 2013, 2019). Deep dissection of the molecular mechanism underlying the leaf senescence may provide a theoretical basis for crop genetic breeding. As leaf senescence involves extensive transcriptional reprogramming, the dynamic activation of transcription factors is considered as a key mechanism that controls the age-dependent expression of thousands of *SAG*s (Woo et al., 2013). Transcriptome profiling has revealed that a number of NAC genes showed enhanced expression during leaf senescence in *Arabidopsi*s, indicating that they play important roles in the senescence process (Kim et al., 2016). Genetic analyses reveal that a number of NAC TFs function as positive (ANAC016, ANAC029/AtNAP, ANAC046, ANAC059/ORS1, and ANAC092/ORE1) (Guo and Gan, 2006; Balazadeh et al., 2010, 2011; Kim et al., 2013; Oda-Yamamizo et al., 2016) or negative (ANAC042/JUB1, ATAF1/ANAC002 and ANAC083/VNI2) regulators of leaf senescence (Yang et al., 2011; Wu et al., 2012; Garapati et al., 2015). Recently, ANAC017, ANAC082, and ANAC090, referred to as a “NAC troika,” are responsible for governing the positive-to-negative regulatory shift and function as negative regulators of leaf senescence in *Arabidopsis* (Kim et al., 2018). Here, our study revealed that NAC TF NAC075 acts as a novel negative regulator in the age-dependent leaf senescence.

Our data demonstrated that NAC075 is a functional *SAG* whose transcription level increases with age (Figure 1A). To this end, we screened knockout lines and generated overexpression transgenic plants to investigate its function in leaf senescence. Loss of function of NAC075 significantly accelerated leaf senescence, whereas overexpression of *NAC075* delayed leaf senescence (Figure 2A), further supporting its negative function in regulating leaf senescence. In addition, overexpression of *NAC075* also evidently suppressed numerous plant hormones or stress-induced leaf senescence. We also found that NAC75 and other NAC TFs may have functional redundancy in regulating leaf senescence and will construct multiple mutants to verify this possibility in the future. RNA-seq profiling analysis revealed that most of the *DEG*s are enriched in response to stimulus, and a large portion of ROS-clearance genes were significantly downregulated in *nac075* mutant plants (Figures 3C,D). Consequently, we observed that the ROS content in *nac075* mutant plants was increased compared with the wild type (Figure 5B). This indicates that NAC075 functions during leaf senescence likely by regulating the expression of ROS-clearance genes. Accordingly, we found that ROS scavenging enzyme *CAT2* is one of the putative target genes of NAC075. H_2_O_2_ is a well-defined inducers of leaf senescence and *CAT2* is a key gene responsible for removing H_2_O_2_ (Vandenabeele et al., 2004; Hieno et al., 2019). Our ChIP-qPCR and EMSA experiments demonstrated that NAC075 bound directly to the *CAT2* promoter, indicating that *CAT2* is a direct target of NAC075 (Figures 4C,D). In addition, *CAT2* overexpression suppresses the early senescence phenotype of *nac075* mutant plants (Figure 5B), providing genetic evidence for the importance of *CAT2* transcription promotion by NAC075 to leaf senescence and ROS accumulation. Based on these results, we conclude that NAC075 suppresses ROS production and leaf senescence by inducing *CAT2* expression. Currently, the upstream TFs regulating age-dependent NAC075 transcription are unclear.

It is reported that increased ROS levels due to decreased antioxidant capacity is highly correlated with leaf senescence (Rogers and Munne-Bosch, 2016). A number of studies have previously reported that NAC TFs regulate leaf senescence by modulating ROS levels, such as JUB1 (ANAC042) (Wu et al., 2012), ATAF1 (ANAC002) (Garapati et al., 2015), ORS1 (ANAC059) (Balazadeh et al., 2011), NTL4 (NAC53) (Lee et al., 2012), ANAC017 (Kim et al., 2018), and ANAC032 (Mahmood et al., 2016). Previous studies reveal that the expressions of JUB1 and ORS1 are induced by H_2_O_2_ (Balazadeh et al., 2011; Wu et al., 2012), while our work found that NAC075 is not response to ROS, suggesting that NAC075 acts as an upstream negative regulator of ROS accumulation but is not induced by ROS.

Based on our data, we proposed a NAC075-CAT2-ROS model to clarify how NAC075 is responsible for delaying leaf senescence (Figure 5E). In this model, *NAC075* transcription is induced by age. Elevated ROS levels lead to PCD and accelerate the senescence process of leaves (Lee et al., 2012; Wu et al., 2012; Rogers and Munne-Bosch, 2016), whereas NAC075 is able to deter the accumulation of ROS by promoting *CAT2* transcription and thereby delay leaf senescence. It is reported that NAC075 is involved in the secondary cell wall formation and the regulation of flowering (Sumire and Nobutaka, 2016). Transcriptome analysis also shows that NAC075 is involved in an array of biotic and abiotic stresses as well as the signal transduction process of plant hormones. Given that the downstream regulatory networks dictated by NAC075 in these processes are still unclear, our finding of the regulatory role of NAC075 in ROS scavenging in leaf senescence offers a potential mechanism for these processes as well.

## Data Availability Statement

The datasets presented in this study can be found in online repositories. The name of the repository and accession number can be found below: National Center for Biotechnology Information (NCBI), https://www.ncbi.nlm.nih.gov/, PRJNA689040.

## Author Contributions

ZL and HG conceived the project and designed the experiments. YS and XX designed part of the experiments. CK carried out most of the experiments. YZ conducted ChIP and EMSA assays. H-LW analyzed the RNA-seq data. ZL and CK wrote the manuscript with input from all co-authors. All authors contributed to the article and approved the submitted version.

## Conflict of Interest

The authors declare that the research was conducted in the absence of any commercial or financial relationships that could be construed as a potential conflict of interest.

## Footnotes

1 http://geneontology.org/

2 https://www.ncbi.nlm.nih.gov/sra/?term=PRJNA689040

## Supplementary Material

Supplementary Figure 1 Transcript level of *SAG12*, *AtNAP* and *ORE1* increases as leaf ages.

Supplementary Figure 2 PCR genotyping of the *nac075* mutants.

Supplementary Figure 3 Overexpression of *NAC075* delayed leaf senescence.

Supplementary Figure 4 The age-dependent pods senescence phenotype of 30-d-old Col-0 and *nac075* mutants.

Supplementary Figure 5 Senescence phenotypes of Col-0, *nac075* mutants and *NAC075ox* plants.

Supplementary Table 1 Primers used in this study.

Supplementary Dataset 1 The differential expressed genes in *nac075* mutant.
