# Corrigendum: Transcription Factor NAC075 Delays Leaf Senescence by Deterring Reactive Oxygen Species Accumulation in *Arabidopsis*

**DOI:** 10.3389/fpls.2021.691607

**Published:** 2021-05-18

**Authors:** Chengcheng Kan, Yi Zhang, Hou-Ling Wang, Yingbai Shen, Xinli Xia, Hongwei Guo, Zhonghai Li

**Affiliations:** ^1^Beijing Advanced Innovation Center for Tree Breeding by Molecular Design, Beijing Forestry University, Beijing, China; ^2^National Engineering Laboratory for Tree Breeding, College of Biological Sciences and Technology, Beijing Forestry University, Beijing, China; ^3^Key Laboratory of Molecular Design for Plant Cell Factory of Guangdong Higher Education Institutes, Department of Biology, Southern University of Science and Technology (SUSTech), Shenzhen, China

**Keywords:** leaf senescence, NAC transcription factor, reactive oxygen species, catalase, *Arabidopsis thaliana*

The original article had errors in Figure 4C and in the caption for **Figure 5B**; see below for details.

**Figure 4:**
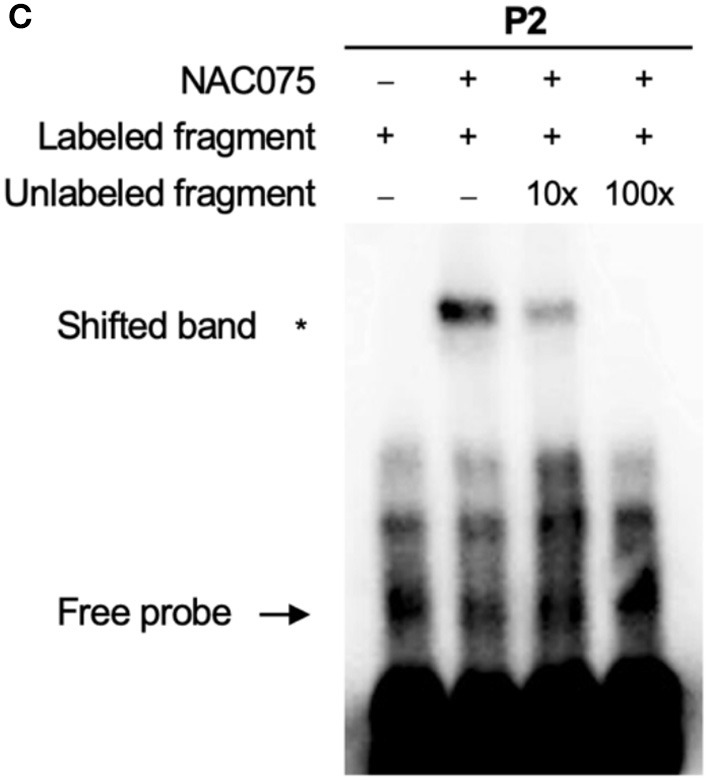
**(C)** EMSA assay of the binding of NAC075 to the *CAT2* promoter *in vitro*. Biotin-labeled probe was used to EMSA experiment and non-labeled fragments were used as competitors. The + and − symbols represent the presence and absence of components. Three biological replicates were performed with similar results.

1. In the original article, there was a mistake in Figure 4C as published. The incorrect electrophoretic mobility shift assay (EMSA) blot was mistakenly introduced during the figure preparation. The corrected Figure 4C appears below.

2. In the original article, there was a mistake in the legend for **Figure 5B** as published. The caption which stated that “DAB staining was used to detect H_2_O_2_ accumulation in the third or fourth leaves of Col-0, *nac075, nac075 CAT2ox*, and *CAT2ox* plants” and that “the brown color represents H_2_O_2_ accumulation” did not also indicate that “NBT staining was used to detect O_2_^−^ accumulation” and that “the blue color represents O_2_^−^ accumulation.” The corrected caption for **Figure 5B** is as follows:

“**Figure 5B**. DAB and NBT staining were used to detect H_2_O_2_ and O_2_^−^ accumulation, respectively, in the third or fourth leaves of Col-0, *nac075, nac075 CAT2ox*, and *CAT2ox* plants. The brown and blue color represent H_2_O_2_ and O_2_^−^ accumulation, respectively. Scale bar, 1 cm.”

The authors apologize for this error and state that this does not change the scientific conclusions of the article in any way. The original article has been updated.

