# Supplementary material for: Transcription Factor NAC075 Delays Leaf Senescence by Deterring Reactive Oxygen Species Accumulation in Arabidopsis
Source: Front Plant Sci. 2021 Feb 24;12:634040. doi: 10.3389/fpls.2021.634040 (PMC7943619; doi:10.3389/fpls.2021.634040)
Supplement: Supplementary Figure 1 — Transcript level of SAG12, AtNAP and ORE1 increases as leaf ages. [file Data_Sheet_1.docx]

**SupplementaryMaterials**

**
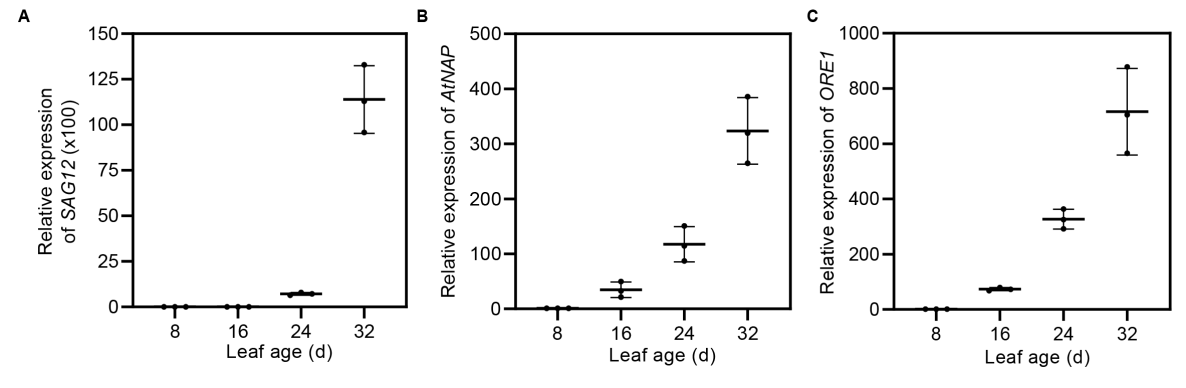
**

**Supplementary Figure 1.**Transcript Level of *SAG12*,*AtNAP* and*ORE1* Increases as Leaf Ages.**(A-C)**qRT-PCR analyses of*SAG12*,*AtNAP* and*ORE1*expression at the indicated leaf age. Data are represented as means ± SD (n = 3).


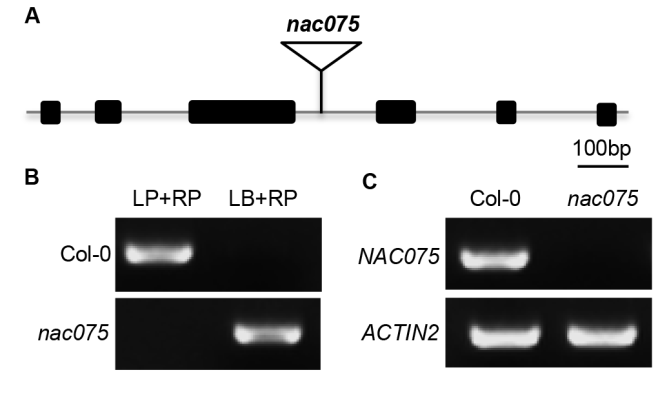


**Supplementary Figure 2.**PCR Genotyping of the *nac075* Mutants. **(A)**Schematic diagram showing the T-DNA insertion in the *nac075* mutants, which is located in the third intron region of *NAC075*.Exons are shown as black boxes and introns as gray lines.**(B)**PCR genotyping of the *nac075*mutants.LP, leftgenomic primer; RP, rightgenomic primer;LB, left border primer of the T-DNA insertion.**(C)**RT-PCR analysis of *NAC075*expression in Col-0 and *nac075* plants. *ACTIN2* was used as an internal control. PCR amplification: *NAC075*, 25 cycles; *ACTIN2*, 28 cycles.

**
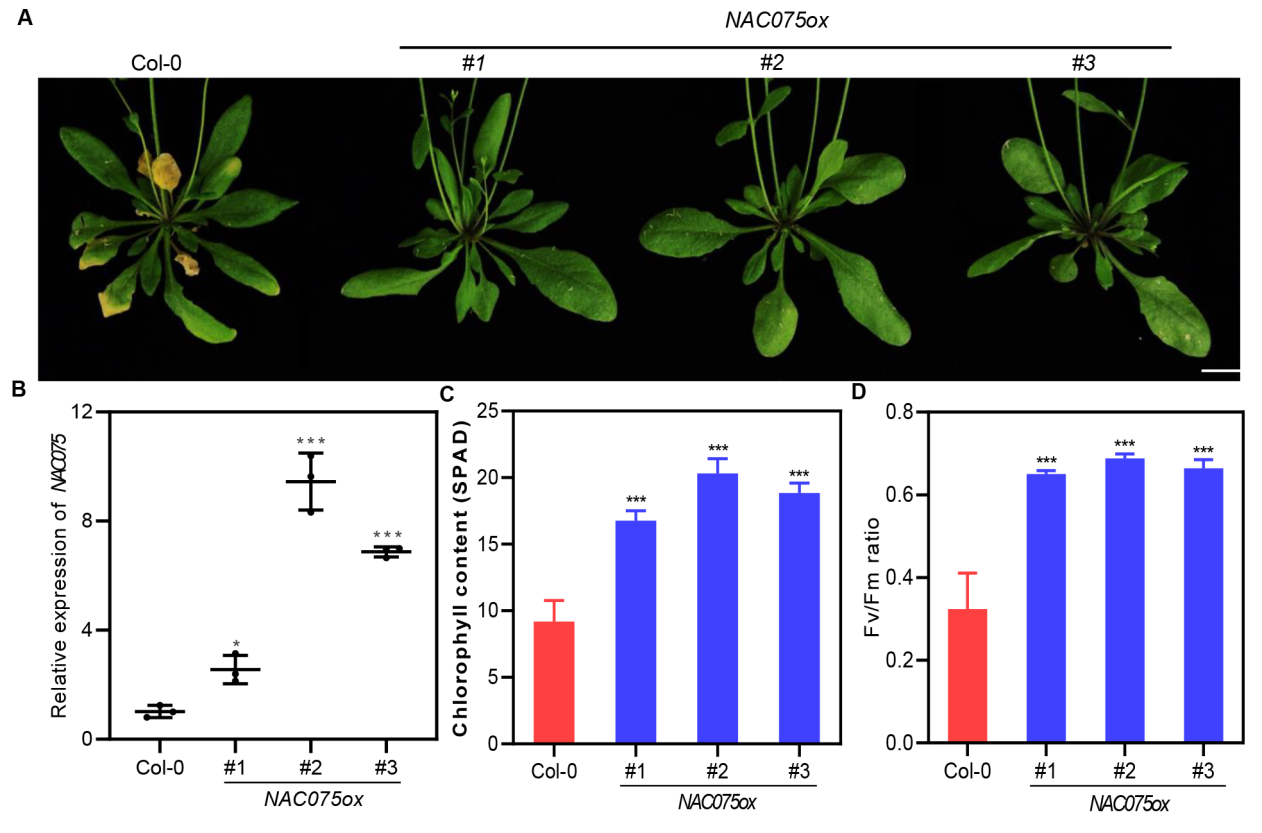
**

**Supplementary Figure3.** Overexpression of *NAC075* Delayed Leaf Senescence**. (A)** Delayed senescence phenotype of *NAC075ox* (#1, #2 and #3) comparedwith Col-0 grown under long-day condition. Scale bar, 1 cm.**(B)** qRT-PCR analyses of *NAC075*expression in *NAC075ox*(#1, #2 and #3). Data are represented as means ± SD (n = 3).Student’s *t*-test, * P <0.1,** P <0.01, *** P <0.001.**(C-D)** Chlorophyll content **(C)** and Fv/Fm **(D)** in *NAC075ox* and Col-0 as leaves age. Error bars indicate SD (n = 3).Student’s *t*-test, *** P <0.001.


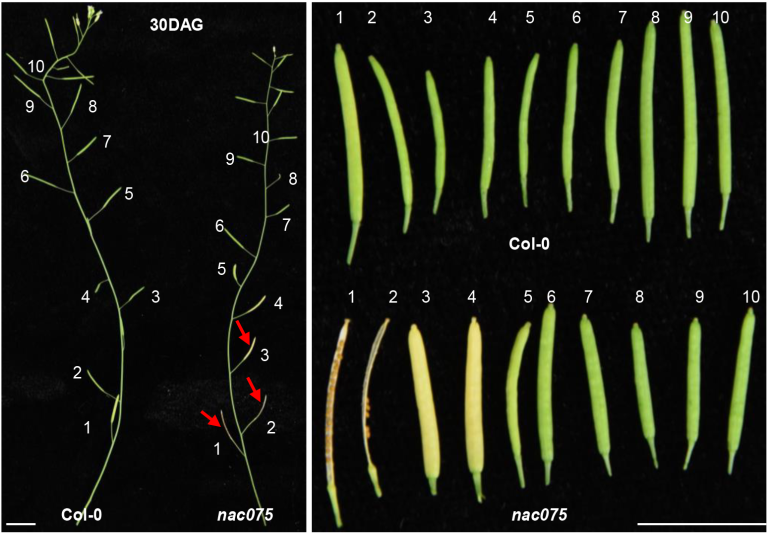


**Supplementary Figure4.**The Age-dependent Pods Senescence Phenotype of 30-d-old Col-0 and *nac075*Mutants.Early onset offruit pods senescencein *nac075* mutation plant compared with wild-type plants grown under long-day condition. Scale bar, 1 cm.


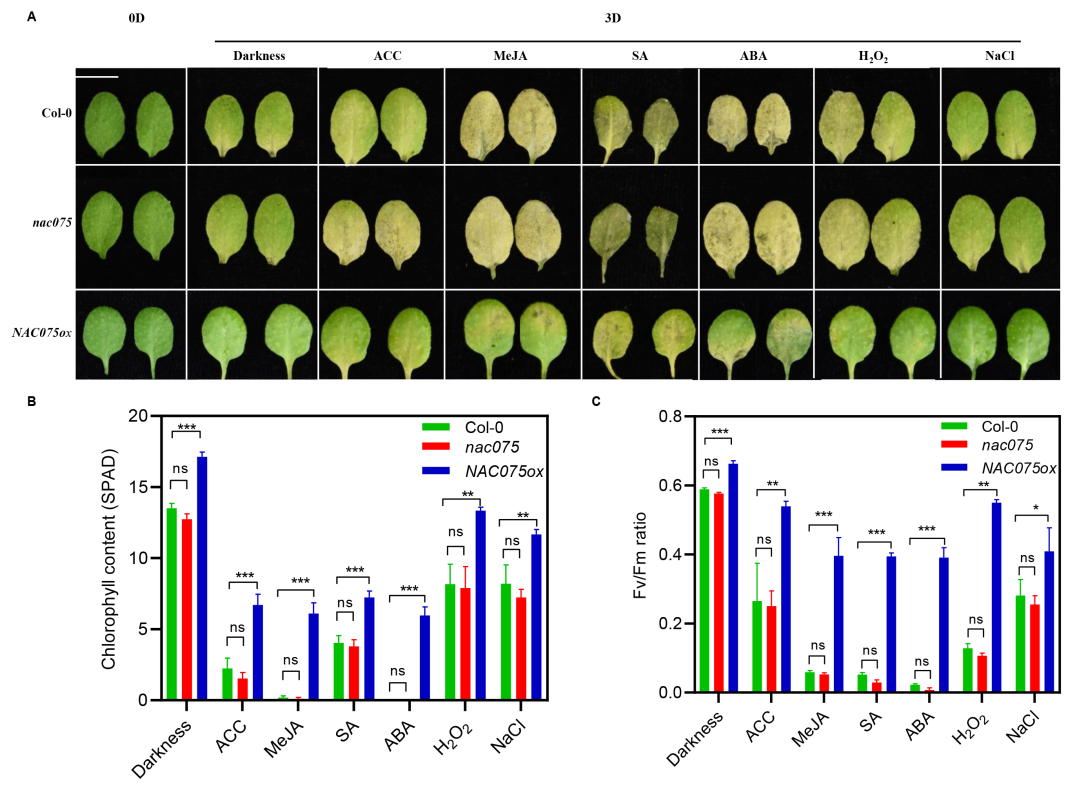


**Supplementary Figure5.**Senescence Phenotypes ofCol-0, *nac075*Mutants and *NAC075ox*Plants.**(A)** The senescence phenotypes ofCol-0, *nac075* mutants and *NAC075ox*Plantsleaves upon treatment with darkness (Mock, 5 mM MES),10 μM ACC, 50 mM MeJA, 1mM SA, 50μM ABA, 10mM H_2_O_2_ or 100mM NaCl.The third or fourth rosette leaveswere detached at 20 DAEand incubated indarkness for 3 days.Scale bar, 1 cm.**(B-C)**Chlorophyll content **(B)** and Fv/Fm **(C)** in the third or fourth rosette leaves of Col-0,*nac075* mutants and *NAC075ox* plants upon treatment with darkness, 10 μM ACC, 50 mM MeJA, 1mM SA, 50μM ABA, 10mM H_2_O_2_ or 100mM NaCl.Error bars indicate SD (n = 6). Student’s *t*-test, * P <0.05, ** P <0.01, *** P <0.001, ns, no significance.

**Supplementary Table 1. Primers Used in This Study.**

| Names | Primer sequence (5'-3') |
| --- | --- |
| **Plasmid construction** | |
| p1391NAC075F | CCGGCGCGCCAAGCTTGGAAATGGGATAAAACGGAGCTCAAATTCT |
| p1391NAC075R | ACTCCTCTTAGAATTCCTCAATCTCGAATATCTTTGATCAACTCTC |
| **Generation of transgenic**  **plants** | |
| pEGADNAC075F | GCGGCCGAATTCCCCGGGATGAACAAGAGTAATCCTGCTGGTT |
| pEGADNAC075R | AAGCTTCTCGAGCCCGGGCCCATGATGATCTTGGTTGTCAGAA |
| pER8NAC075F | GCTAGTCGACTCTGCCATGAACAAGAGTAATCCTGCTGGTTCGGTG |
| pER8NAC075R | TTGTAGTCACTAGTTAACCCCCATGATGATCTTGGTTGTCAGAAGAGTC |
| **qRT-PCR** | |
| SAG12F | CAGCTGCGGATGTTGTTG |
| SAG12R | CCACTTTCTCCCCATTTTG |
| CAT2F | CTTCTCCTATGCCGATACTCAG |
| CAT2R | CAATAATGCACCTCTCACGTTT |
| NAC075F | CATTCAACATAAGCCAGCCTAC |
| NAC075R | GTTGCTGTTGTGTCTGGTAATT |
| AtNAPF | TAACGTAGGTGTCAAGAAAGCT |
| AtNAPR | GCTTTACGTGAATCATGGAGAC |
| ORE1F | ACAGCTAAGAACGAATGGGTTA |
| ORE1R | CCATTCGGTTAATGTGTGGATC |
| UBC21F | TCAAATGGACCGCTCTTATC |
| UBC21R | CACAGACTGAAGCGTCCAAG |
| **ChIP assays** | |
| TUB2F | GAGCCTTACAACGCTACTCTGTCTGT |
| TUB2R | ACACCAGACATAGTAGCAGAAATCA |
| CAT2-P1F | TTCAATTATGCCTTGAGAATGTGCT |
| CAT2-P1R | CCAAATAAATTGATTGTCTGCGGTA |
| CAT2-P2F | CATCTCATTGTTTCTTCAGCACATT |
| CAT2-P2R | TGCCGAATTCTATTGGTTATAAAGA |
| **EMSA** | |
| pET32a-NAC075F | GCCCGGATCCGAATTCATGAACAAGAGTAATCCTGCTGG |
| pET32a-NAC075R | GGTGGTGGTGCTCGAGACCACCTATAGCGTTCAAGCTCG |
